# League of psychiatry and mental health of a brazilian university: Promoting mental health in COVID-19 times

**DOI:** 10.1192/j.eurpsy.2021.810

**Published:** 2021-08-13

**Authors:** L. Floriano, P. Oliveira, B. Cardoso, E. Locaste, N. Nabozny, F. Ferreira

**Affiliations:** 1 Nursing And Public Health, State University of Ponta Grossa, Ponta Grossa, Brazil; 2 Medicine, State University of Ponta Grossa, Ponta Grossa, Brazil

**Keywords:** Health promotion, mental health, COVID-19

## Abstract

**Introduction:**

Due to the current global background of the COVID-19 pandemic, mental health is an important factor to be promoted. In spite of the subjectivity of the psychological impact brought by this pandemic, the population has undergone several sudden and meaningful changes in psychic integrity. Therefore, the League of Psychiatry and Mental Health of a Brazilian public university emerges with the aim of complementing the curriculum of Psychiatric Medicine, along with promoting mental health inside and outside the university.

**Objectives:**

Hold online events and disseminate informative material to help students, mental health professionals and general community interested in the topic.

**Methods:**

The League did a member recruitment with academics from different health areas, who prepared and published booklets and folders with informations promoting mental health. And also, promoted speeches on online platforms from May 2020 to October 2020 with psychiatrists, psychologists and renowned professionals in Brazil.

**Results:**

Eighteen lectures were held on topics such as “Grief in the pandemic and its implications in mental health”; “Preventing suicide in the pandemic”, among others, that had a relevant role for those who were in a vulnerable emotional state at the time. As for publications, a national reach was possible, which served as a source for the cultivation of a good psychic health to face the pandemic.

**Conclusions:**

More than 13.600 people participated in the promoted proposals, In addition to providing positive feedbacks to the League, with the improvement of knowledge in the field of Psychiatry and Mental Health, reaching the proposed objectives.

